# Evaluation of Memorial Sloan‐Kettering Cancer Center and International Extranodal Lymphoma Study Group prognostic scoring systems to predict Overall Survival in intracranial Primary CNS lymphoma

**DOI:** 10.1002/brb3.928

**Published:** 2018-02-05

**Authors:** Guro Jahr, Michele Da Broi, Harald Holte, Klaus Beiske, Torstein R. Meling

**Affiliations:** ^1^ Department of Neurosurgery Oslo University Hospital Oslo Norway; ^2^ Faculty of Medicine Institute of Clinical Medicine University of Oslo Oslo Norway; ^3^ Department of Oncology Oslo University Hospital Oslo Norway; ^4^ Department of Pathology Oslo University Hospital Oslo Norway

**Keywords:** chemotherapy, lymphoma, oncology, Overall Survival, Primary CNS lymphoma, prognostic factors, surgery

## Abstract

**Objectives:**

To evaluate the validity of Memorial Sloan‐Kettering Cancer Center (MSKCC) and International Extranodal Lymphoma Study Group (IELSG) prognostic scoring systems for Overall Survival (OS) in intracranial Primary CNS lymphoma (PCNSL) of all patients diagnosed at a single center.

**Material and Methods:**

Pretreatment clinical factors including tumor characteristics and histology, treatment, and survival of PCNSL patients with diagnostic biopsies over a 12‐year period (2003–2014) were retrieved from a prospective database at Oslo University Hospital.

**Results:**

Seventy‐nine patients with intracranial PCNSL were identified. The female:male ratio was 1:1.63 and the median age was 65.3 years [range 18.9–80.7]. Involvement of deep brain structures was shown in 63 patients. Six patients were MSKCC risk group 1, 35 patients were in risk group 2, and 38 patients were in risk group 3. International Extranodal Lymphoma Study Group scores were <2 in 17 patients (22%). After a median follow‐up of 70.5 months, 55 patients were dead. Median OS was 16.4 months [range 0.2–157.7]. Age, sLDH by recursive partitioning analysis (RPA), Eastern Cooperative Oncology Group score (ECOG), lesion size, involvement of deep brain structures, IELSG score, and MSKCC score were significant factors for OS in univariate analysis. Multivariate analysis confirmed the significance of age (*p* < .05), sLDH by RPA (*p* < .005), ECOG (*p* < .05), and deep brain structure involvement (*p* < .05) for OS. The six‐tiered IELSG scores had to be dichotomized according to RPA analysis into <2 and ≥2 in order to have prognostic value. In contrast, when using the three‐tiered MSKCC, three distinct risk groups were identified.

**Conclusions:**

Our study failed to verify the IELSG, but validated the use of MSKCC for prognostication of OS in intracranial PCNSL.

## 1 INTRODUCTION

Despite the uncertainty in survival prediction, existing prognostic tools can facilitate clinical decision making. Even though several prognostic scoring systems have been proposed, stratification of Primary CNS lymphoma (PCNSL) patients is still challenging for clinicians. A well‐defined prognostic score should be easy to calculate without including parameters complicated to obtain. Furthermore, it should allow a clear separation of patients into risk groups and have a high predictive value. A well‐established scoring system could be used for risk‐tailored therapeutic strategies and risk‐adjusted follow‐up as well as a basis for comparing treatment results in clinical studies.

The International Extranodal Lymphoma Study Group (IELSG) designed in 2003 a scoring system to identify survival predictors useful for distinguishing risk groups in immunocompetent patients with PCNSL. The score is based on five parameters, namely age >60 years, elevated serum LDH, Eastern Cooperative Oncology Group score (ECOG) ≥2, involvement of deep brain structures, and raised cerebrospinal fluid (CSF) protein levels. Each parameter of IELSG can be either favorable (0) or unfavorable (1) and based on the final sum, three different risk groups can be distinguished (Ferreri et al., 2003).

A simpler score was proposed in 2006 by the Memorial Sloan‐Kettering Cancer Center (MSKCC) that consists of only patient age and Karnofsky performance status (KPS) (Abrey et al., 2006). In contrast to the IELSG score, the MSKCC uses 50 years as age cutoff. In patients older than 50 years, the most significant variable for survival was KPS >70. Furthermore, neither CSF protein level nor sLDH is required to calculate MSKCC. According to the MSKCC, patients are classified into three risk groups, namely: age ≤50 years; age >50 years and KPS ≥70; age >50 years and KPS <70 (Abrey et al., 2006).

The prognostic value of the IELSG score was recently confirmed in a prospective trial (Ferreri et al., 2006). However, Ferreri and Reni (2005) found statistically significant differences between some of the IELSG groups, consistent with Bessell et al. (2004) observation. These observations have raised doubts about the reliability of this model. Conversely, other publications have reported no prognostic discrimination by the MSKCC score (Schorb et al., 2013; Wieduwilt et al., 2012). We therefore wanted to evaluate and compare the IELSG and MSKCC prognostic scoring systems for Overall Survival (OS) in PCNSL in our patient cohort of consecutively diagnosed patients from a single center.

## 2 MATERIAL AND METHODS

### 2.1 Clinical setting

Oslo University Hospital (OUH) is a tertiary referral center with a catchment area of approximately 3 million inhabitants (56% of the Norwegian population).

### 2.2 Patient cohort

Prospective databases for brain tumors at the Department of Neurosurgery and CNS lymphomas at the Department of Oncology at OUH were searched to identify the patients. Inclusion criteria were histologically verified intracranial PCNSL between 2003 and 2014. Exclusion criteria were lymphomas in the intraorbital space, epidural space, and intraspinal lesions.

### 2.3 Patient‐related variables

The medical records of patients were reviewed retrospectively to record parameters of interest not included in the databases. We recorded age, sex, time from symptoms to diagnosis, time of surgery, time of death, KPS, ECOG score, LDH, immune status, MSKCC, and IELSG. Time of diagnosis was set as time of surgery. Cutoff for sLDH is age‐dependent: For patients ≤69 years, sLDH < 205 U/L is regarded as normal, while patients >69 years have a cutoff of <255 U/L. Immunocompromise was defined as EBV+, HIV+, TBC+, or organ transplantation.

### 2.4 Tumor‐related variables

A histopathological diagnosis of PCNSL was made by a consultant pathologist at presentation. All cases were formally reexamined by a dedicated hematology pathologist.

T1‐weighted contrast‐enhanced MRI images were reviewed by the first and senior authors. The variables recorded were as follows: tumor location, involvement of deep brain structures (defined as periventricular regions, basal ganglia, corpus callosum, brainstem, and/or cerebellum), maximum visible diameter of the lesion, and number of lesions.

### 2.5 Treatment

Eligible patients (excluding the elderly and patients with reduced renal function) were treated according to a MSKCC protocol (Abrey, Yahalom, & DeAngelis, 2000) with the addition of rituximab since 2010 or for the period May 2007–October 2010 according to a Nordic protocol (Pulczynski et al., 2015), both with high‐dose methotrexate as a cornerstone in the treatment. Patients not eligible were treated according to doctor's choice, that is, with radiotherapy with or without corticosteroids only. Treatment and survival related to treatment will be reported separately, and details are not given in this manuscript.

### 2.6 Outcome

All patients underwent multidisciplinary follow‐up for the assessment of outcomes. Vital status and date of death were retrieved from Norwegian Population Registry at 19.12.2016. OS was calculated from time of diagnosis to time of death or censoring.

### 2.7 Statistics

Univariate statistics were calculated without assuming a Gaussian distribution using Wilcoxon's test when the variable was continuous. With categorical variables, univariate statistics were calculated using Fisher's exact test. Survival curves were generated using the Kaplan–Meier estimator, and the log‐rank test was used to compare different survival curves. Prognostic factors were identified using the Cox proportional hazards regression model. Odds ratios (OR) were calculated to estimate the strength of association between OS and prognostic factors as binary data values. Recursive partitioning analysis (RPA) was used to search all possible splits between the variable values seeking to maximize an information measure difference between the two nodes yielding a RPA tree for prognostic factors. In our analysis, alpha for stopping the growth of the tree was set at .05, and log‐rank scores were used for the censored data. Descriptive statistics were reported as a mean with a 95% confidence interval (CI) or a median with a range, as appropriate. A *p*‐value <.05 was considered significant. For all statistical analysis, the software program JMP (version 9.03, SAS Institute Inc. RRID: SCR_014242) was used.

### 2.8 Ethics

The study was approved by the Data Protection Office at OUH (2015/16840).

## 3 RESULTS

### 3.1 Patient characteristics

The female:male ratio was 1:1.63, with 30 females (38%) and 49 males (62%). The median age of the population was 65.3 years [range 18.9–80.7]. Immunocompromise was present in 10 patients (13%). Twenty‐six (33%) patients had elevated sLDH, while 44 patients (56%) had normal sLDH. Eighteen patients (23%) had an ECOG score of 0 (Table 1).

**Table 1:** Patient and tumor characteristics

	*n*	%
Sex		
M	49	62
F	30	38
Age		
<50 years	6	8
≥50 years	73	92
≥60 years	55	70
≥70 years	26	33
Age (RPA)		
<52.6 years	12	15
≥52.6 years	67	85
Immunodeficiency		
Yes	10	13
No	69	87
sLDH		
Elevated	13	17
Not elevated	57	72
NA	10	11
ECOG		
0	18	23
≥1	61	77
KPS		
≥70	39	49
<70	40	51
IELSG score		
0	5	6
1	12	15
2	21	27
3	28	35
4	4	5
5	0	0
NA	9	11
MSKCC score		
Risk group 1	6	8
Risk group 2	35	44
Risk group 3	38	48
Multiplicity		
1	32	40
2–4	38	48
≥5	7	9
NA	2	3
Size		
≤30 mm	23	29
30–50 mm	30	38
≥50 mm	21	27
NA	5	6
Size (RPA)		
<64.9 mm	69	83
≥64.9 mm	5	6
Deep brain structures		
Not involved	16	20
Involved	63	80

ECOG, Eastern Cooperative Oncology Group; IELSG, International Extranodal Lymphoma Study Group; MSKCC, Memorial Sloan‐Kettering Cancer Center; RPA, recursive partitioning analysis; KPS, Karnofsky performance status.

All patients included in this study underwent surgery. Craniotomy with resection was performed in 32 patients (41%), while all other patients received biopsies. Twenty‐two patients (28%) underwent a stereotactic biopsy, while 20 (25%) received an open biopsy, and 5 (6%) an endoscopic biopsy.

Fifty‐seven patients (72%) were treated with chemotherapy according to either the Nordic protocol or the MSKCC protocol, but without radiotherapy for patients achieving a CR on chemotherapy, and 22 patients (28%) received only radiotherapy (*n* = 7, 9%) or palliative treatment (*n* = 15, 19%).

### 3.2 Tumor characteristics

Thirty‐two patients (40%) had one lesion, 38 patients (48%) had 2–4 lesions, while 7 patients (9%) were diagnosed with ≥5 lesions. The mean size of lesions was 40.3 mm (CI 43.7–36.9 mm). In 30 patients (38%), the greatest diameter was between 30 and 50 mm. Twenty‐three patients (29%) had lesions ≤30 mm in diameter, while 21 patients (27%) presented diameters ≥50 mm. Involvement of deep brain structures was shown in 63 patients (80%) (Table 1). Most frequently, lesions were localized in the frontal lobe (*n* = 33, 42%), temporal lobe (*n* = 27, 34%), parietal lobe (*n* = 23, 29%), and corpus callosum (*n* = 21, 27%). Fifty‐four patients (68%) had periventricular lesions.

### 3.3 Overall Survival

**Figure 1:**
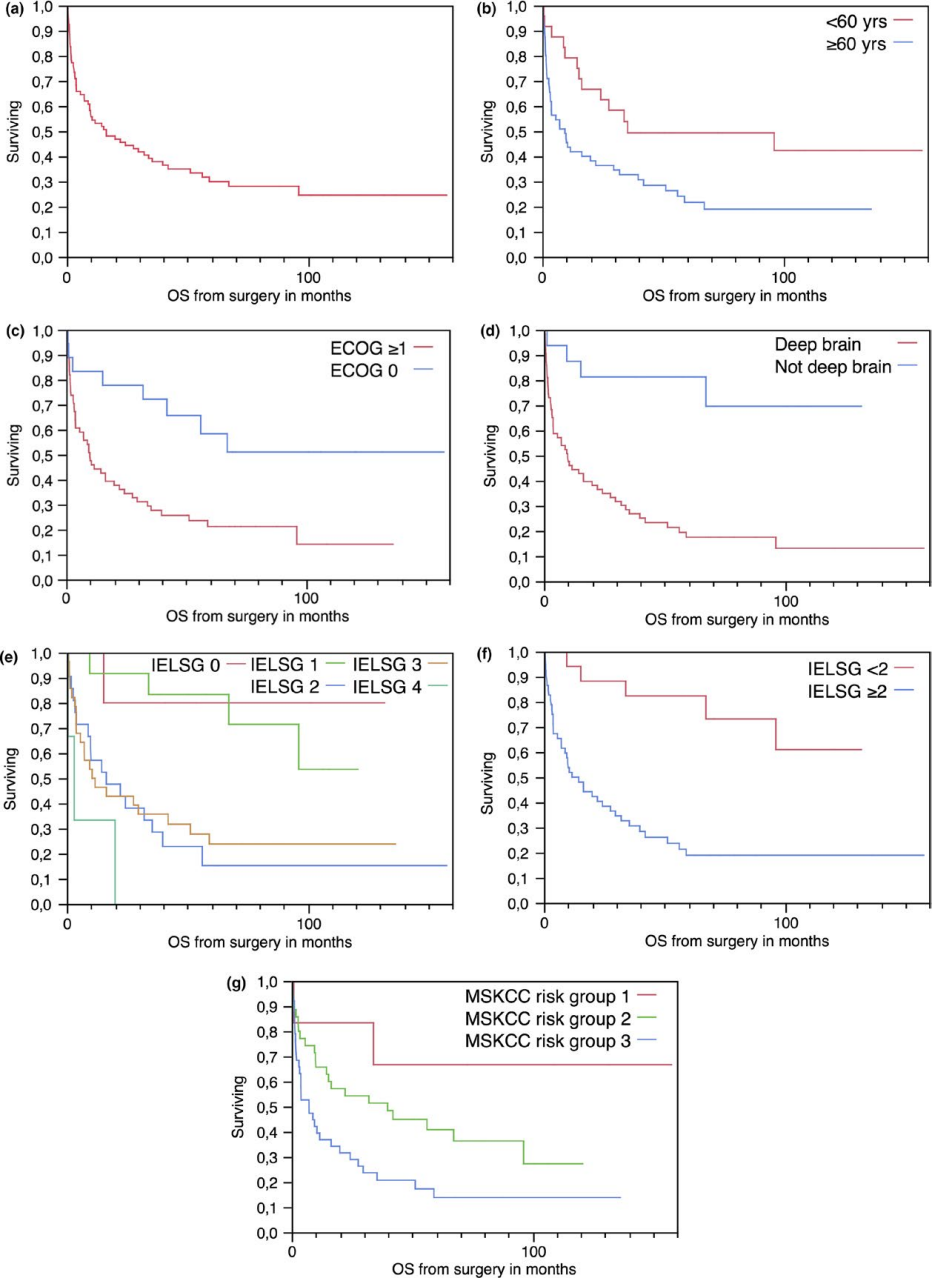
Overall Survival. (a): Median OS. (b): OS by age at surgery. (c): OS by ECOG dichotomized by RPA. (d): OS by deep brain involvement. (e): OS by IELSG score. (f): OS by IELSG score dichotomized by RPA. (g): OS by MSKCC score. ECOG, Eastern Cooperative Oncology Group; IELSG, International Extranodal Lymphoma Study Group; MSKCC, Memorial Sloan‐Kettering Cancer Center; OS, Overall Survival; RPA, recursive partitioning analysis

At the end of the study, after a median follow‐up of 70.5 months [range 33.3–157.7], 55 patients (70%) were dead. Median OS was 16.4 months [range 0.2–157.7] (Table 2; Figure 1a).

**Table 2:** Prognostic factors for OS

	*n*	Overall Survival in months (median [range])	*p*‐value for OS
	79	16.4 [0.2–157.7]	
Sex			
Female	30	38.0 [0.4–131.9]	NS
Male	49	14.4 [0.2–157.7]	
Age			
<50 years	6	90.9 [0.9–157.7]	NS
≥50 years	73	14.4 [0.2–136.5]	
≥60 years	55	9.4 [0.2–136.5]	<.05
≥70 years	26	3.8 [0.4–69.4]	<.05
Age (RPA)			
<52.6 years	12	98.7 [0.9–157.7]	<.05
≥52.6 years	67	10.0 [0.2–136.5]	
Immunodeficiency			
Yes	10	14.4 [0.5–157.7]	NS
No	69	16.4 [0.2–136.5]	
sLDH (RPA)			
Elevated	13	5.6 [0.5–106.3]	<.005
Not elevated	57	35.4 [0.7–157.7]	
ECOG			
0	18	54.9 [0.7–157.7]	<.01
≥1	61	9.8 [0.2–136.5]	
KPS			
≥70	39	39.8 [0.4–157.7]	<.01
<70	40	7.2 [0.2–136.5]	
Multiplicity			
1	32	22.2 [0.2–157.7]	NS
2–4	38	16.4 [0.4–136.5]	
≥5	7	3.9 [0.5–120.8]	
Size (RPA)			
<64.9 mm	69	24.3 [0.4–157.7]	<.005
≥64.9 mm	5	3.0 [0.2–9.8]	
Deep brain structures			
Involved	63	9.8 [0.2–157.7]	<.0005
Not involved	16	60.1 [1.2–131.9]	
IELSG score			
<2	17	67.3 [9.4–131.9]	<.01
≥2	53	11.7 [0.5–157.7]	
MSKCC score			
Risk group 1	6	90.9 [0.9–157.7]	<.01
Risk group 2	35	38.5 [0.4–120.8]	
Risk group 3	38	7.2 [0.2–136.5]	

ECOG, Eastern Cooperative Oncology Group; IELSG, International Extranodal Lymphoma Study Group; KPS, Karnofsky performance status; MSKCC, Memorial Sloan‐Kettering Cancer Center; NS, not significant; OS, Overall Survival; RPA, recursive partitioning analysis.

Recursive partition analysis identified age as the most relevant prognostic factor for OS and identified a split at 52.6 years of age. Patients <52.6 years had a median OS of 98.7 months [range 0.9–157.7], while patients ≥52.6 had 10.0 months [range 0.2–136.5] (*p* < .05) (Table 2). The OR for patients ≥52.6 years was 4.5 [range 1.2–16.0] (*p* < .05) (Table 4). Patients <60 years showed a median OS of 34.7 months [range 0.7–157.7] versus 9.4 months [range 0.2–136.5] for those ≥60 years (*p* < .05) (Figure 1b, Table 2). Regarding sLDH, RPA analysis identified a split at 309 U/L. With respect to lesion size, RPA identified the cutoff at 64.9 mm.

The following factors were significant for OS according to univariate analysis: age at surgery (Figure 1b), sLDH by RPA cutoff, ECOG (Figure 1c), lesion size, deep brain involvement (Figure 1d), IELSG (Figure 1e), IELSG dichotomized by RPA (Figure 1f), and MSKCC (Figure 1g) (Table 2). Sex, immunostatus, sLDH, and multiplicity were not significant prognostic factors for OS (Table 2).

Multivariate analyses confirmed the significance for OS of the following variables: age at surgery (*p* < .05), sLDH by RPA cutoff (*p* < .005), ECOG (*p* < .05), and involvement of deep brain structures (*p* < .05) (Table 3).

**Table 3:** Multivariate analysis of prognostic factors for OS

	*p*‐value
Age	<.05
sLDH (RPA)	<.005
ECOG	
ECOG 0	<.05
ECOG 1	<.005
ECOG 2	<.005
ECOG 3	<.01
ECOG 4	<.05
Deep brain structures	<.05

ECOG, Eastern Cooperative Oncology Group; OS, Overall Survival; RPA, recursive partitioning analysis.

The OR for IELSG ≥2 was significant with 9.0 [range 2.6–30.8]. Conversely, the OR was 3.4 [range 0.5–21.1] for patients in MSKCC risk group 2 and 10.7 [range 1.6–71.9] for risk group 3 (Table 4).

**Table 4:** Odds ratios of prognostic factors for OS

	*n*	Overall Survival OR [CI 95%]	*p*‐value for OS
Age (RPA)			
<52.6 years	12	1.0	<.05
≥52.6 years	67	4.5 [1.2–16.0]	
sLDH (RPA)			
Not elevated	57	1.0	NS
Elevated	13	3.2 [0.7–15.9]	
ECOG			
0	18	1.0	<.01
≥1	61	4.6 [1.5–14.1]	
KPS			
≥70	39	1.0	<.05
<70	40	3.3 [1.5–14.1]	
Deep brain structures			
Not involved	16	1.0	<.0001
Involved	63	14.2 [3.8–52.3]	
IELSG score			
<2	17	1.0	<.01
≥2	53	9.0 [2.6–30.8]	
MSKCC score			
1	6	1.0	
2	35	3.4 [0.5–21.1]	NS
3	38	10.7 [1.6–71.9]	<.05

ECOG, Eastern Cooperative Oncology Group; IELSG, International Extranodal Lymphoma Study Group; KPS, Karnofsky performance status; MSKCC, Memorial Sloan‐Kettering Cancer Center; NS, not significant; OS, Overall Survival; OR, odds ratio; RPA, recursive partitioning analysis; PS, prognostic score.

## 4 DISCUSSION

Despite improvements in chemotherapy protocols and more sensitive imaging for early diagnoses, PCNSL still has a dismal prognosis (Norden, Drappatz, Wen, & Claus, 2010). In our study of 79 patients with intracranial PCNSL, median OS was 16.4 months (Figure 1a, Table 2). In a Swedish study, also with inclusion of all patients from a defined area from a similar time period, a median OS of only 4 months is reported (Enblad et al., 2017). Other authors such as Korfel et al. (2015) and Ghesquières et al. (2010) reported longer survival rates from prospective studies. However, half of our patients were in MSKCC risk group 3 and only six (8%) were in MSKCC risk group 1 (Table 1). The increased fraction of high‐risk patients negatively impacted our survival rates. Indeed, when comparing OS for specific risk groups, our results are in accordance with the literature.

The most important prognostic factor was patient age. This is consistent with the literature (Fraser, Gruenberg, & Rubenstein, 2015). In fact, both IELSG and MSKCC prognostic models include patient age (Abrey et al., 2006; Ferreri et al., 2003). The cutoff identified using RPA was 52.6 years in our data, similar to that used in MSKCC (Abrey et al., 2006) and lower than the cutoff used in IELSG, namely 60 years (Ferreri et al., 2003). However, old age and high ECOG score should not necessarily be considered as exclusion criteria for treatments with curative intent, as they tend to lose their prognostic value after treatment (Gavrilovic, Hormigo, Yahalom, DeAngelis, & Abrey, 2006; Ghesquières et al., 2010, 2012). Indeed, also elderly fit patients benefit from high‐dose therapy given with a median dose intensity (Kasenda et al., 2015). Furthermore, ECOG is dependent on neurological status and hence affected by steroid treatment (Ghesquières et al., 2012).

The other main prognostic factors that were significant in our multivariate analysis were sLDH, ECOG, and deep brain location. According to Ghesquières et al. (2012), sLDH was a solid and durable predictor of OS, while performance status was time‐dependent and lost the prognostic value after 6 months. On the other hand, the OR calculated for sLDH was not significant in our analysis, while the OR for ECOG was significant. Deep brain location together with patient age was the soundest prognostic factors in this study (Figure 1).

It is remarkable, how immunological status is no longer a prognostic factor for PCNSL (Table 2) and cannot be an exclusion criterion for therapy. This is probably because of effective new antiretroviral treatments and immunocompromised patients represented only 13% for our study population. This confirms the findings by Haldorsen et al. (2008) that the number of AIDS‐related PCNSL is decreasing.

Our study failed to verify the IELSG for prognostication of OS (Figure 1e) as the IELSG score had to be dichotomized according to an RPA analysis into <2 and ≥2 in order to have prognostic value (Figures 1f). Other authors grouped together 0–1, 2–3, and 4–5 classes of IELSG (Fraser et al., 2015). Furthermore, calculation of IELSG also includes CSF protein levels which are not always available for every patient. There is a high rate of missing values for this parameter both in our study and other retrospective cohorts (Ghesquières et al., 2010; Schorb et al., 2013). Primary CNS lymphoma patients often present with space‐occupying intracranial lesions with perifocal edema and presumed raised intracranial pressure. Therefore, lumbar punctures are often not performed in routine clinical practice before initiation of therapy, resulting in a substantial proportion of patients with incomplete IELSG scores. Certainly, this lack of simplicity is a limitation of that score. In fact, we could not calculate the IELSG score for 9 of our patients (11%), while calculation of the MSKCC score was always possible (Table 1).

Memorial Sloan‐Kettering Cancer Center requires only patient age and performance status and when used, three distinct risk groups were identified (Figure 1g). The OS was 90.9, 38.5, and 7.2 months for MSKCC group 1, 2, and 3, respectively (Table 2).

Our cohort represents one of the larger unselected series available in the literature. Furthermore, the quality of our data collection with complete follow‐up for all patients is high. Moreover, due to the centralized administration of chemotherapy, our data on treatment are homogeneous for defined time periods.

Cerebrospinal fluid protein level was not available for the majority of patients; thus, we could not include that parameter in when calculating the IELSG score. This is potentially a limiting factor, even though Ghesquières et al. (2012) and Kiewe, Fischer, Martus, Thiel, and Korfel (2010) observed no prognostic impact of CSF protein levels in PCNSL prognosis. Korfel et al. (2012) asserted that CSF protein levels can be related to the meningeal dissemination of PCNSL, but this complication does not seem to be significant for the prognosis.

Due to the retrospective nature of this study, our data were not always complete. The IELSG score was not calculated in nine cases because of the lack of sLDH values. Furthermore, both the pretherapeutic and therapeutic characteristics of our cohort were heterogenous. Indeed, our patients were treated according to two different chemotherapy protocols and 13% of them were immunocompromised.

Although other authors have confirmed the validity of these prognostic scores before (Fraser et al., 2015; Ghesquières et al., 2012), to our knowledge, this is the first direct comparison of the IELSG and MSKCC prognostic scoring systems for intracranial PCNSL. We failed to validate the IELSG prognostic scoring system in its original form for OS of intracranial PCNSL, although patient age, ECOG > 1, deep brain involvement, and sLDH were independent predictors of OS. In contrast, MSKCC identified three distinct risk groups and its ease of use makes it preferable.

## CONFLICT OF INTEREST

None declared.
